# Clinical Efficacy and Safety of Stem Cell-Based Therapy in Treating Asherman Syndrome: A System Review and Meta-Analysis

**DOI:** 10.1155/2020/8820538

**Published:** 2020-12-19

**Authors:** Yiming Zhao, Qifan Luo, Xiao Zhang, Yafei Qin, Jingpeng Hao, Dejun Kong, Hongda Wang, Guangming Li, Xiangying Gu, Hao Wang

**Affiliations:** ^1^Department of General Surgery, Tianjin Medical University General Hospital, Tianjin, China; ^2^Tianjin General Surgery Institute, Tianjin Medical University General Hospital, Tianjin, China; ^3^Department of Radiation and Medical Oncology, Zhongnan Hospital of Wuhan University, Wuhan, China; ^4^Department of Hepatobiliary and Pancreatic Surgery, Henan Provincial People's Hospital, Zhengzhou, China; ^5^Department of Anorectal Surgery, The Second Hospital of Tianjin Medical University, Tianjin, China; ^6^Department of Gynecology and Obstetrics, Tianjin Medical University General Hospital, Tianjin, China

## Abstract

Asherman's Syndrome (AS) is an uncommon, acquired, and refractory gynecological disorder. Current treatment was still limited, and stem cell-based therapy has been proposed as a novel strategy for management of AS. Here, we conducted a meta-analysis of self-controlled clinical trials to assess the effectiveness and safety of stem cell-based therapy in Asherman syndrome patients who have failed in conventional treatment. We systematically searched PubMed, Embase, Cochrane, and Web of Science database (published up to October 3, 2020). Our main evaluation outcomes were menses improvement, endometrial thickness changes, pregnancy outcome, and side effects. All analyses were performed by using RevMan5.4 software. 427 studies were identified, eight of which were eligible and included in our analysis. Stem cell combined hormone therapy achieved a higher likelihood of improving menstruation (risk ratio [RR] 22.43, 95% CI: 8.03 to 62.68, *P* < 0.00001), an enhancement of pregnancy outcome (risk ratio [RR] 11.1, 95% CI: 3.58 to 34.38, *P* < 0.0001), and a mean increase of 3-month endometrial thickness (standardized mean difference [SMD] 2.43, 95% CI: 1.72 to 3.13, *P* < 0.00001). Subgroup analysis also indicated that 6-month and 9-month endometrial thickness increased significantly with the stem cell-based treatment. Moreover, no obvious and severe adverse reactions were observed during the process of stem cell therapy. There were 3 patients (3.57%) reported with lost appetite, mild gastritis, vomiting, or abdominal cramps, whereas, these symptoms relieved subsequently. This meta-analysis systematically reviewed and synthesized the outcomes of stem cell-based therapy in treating Asherman syndrome, which suggest that stem cell and hormone combination therapy was safe and more effective in improving menstruation duration, pregnancy outcome, and endometrial thickness. However, further trials with large sample sizes are needed to establish more solid evidence for administrating this therapy in clinic.

## 1. Background

Asherman's Syndrome (AS) is defined by the obliteration of the uterine cavity and thin endometrium, which results from partial or complete fusion of opposing uterine wall, also referred as intrauterine adhesion (IUA) [1, 2]. AS patients often complain of hypomenorrhea, amenorrhea, infertility, and adverse pregnancy outcome [3]. Curettage of endometrium shortly after pregnancy which is mainly responsible for the development of IUA. Therefore, the clinical goal for treating AS is to recover the uterine cavity and restore the endometrium function [4].

So far, hysteroscopic adhesiolysis has been considered as a primary choice for the treatment of intrauterine adhesion [5]. However, surgical therapy cannot cure over half of the AS patients and fail to achieve successful pregnancy in infertile women caused by IUA [6]. Hormone therapy is recommended to maintain high estrogen level, which can promote the process of endometrium healing [3]. Nevertheless, hormone therapy is inadequate to restore the endometrial function [7]. Under this circumstance, stem cell therapy can provide an alternative way to reconstruct endometrium and lead to a successful reproductive outcome [8].

Adult stem cells are undifferentiated cells, found throughout the body after development and with the ability in proliferation and multiple differentiation, which makes it possible to initiate endometrial restoration [9, 10]. Evidence has suggested that transplantation of various kinds of adult stem cells could incorporate into endometrium and differentiate into endometrial epithelial, stromal, or endothelial cells, thus reconstructing endometrial tissues [11–14]. However, to date, no system review and meta-analysis have evaluated the efficacy and safety of clinical trials for stem cell treatment of AS patients.

Therefore, we conduct this present meta-analysis to analyze clinical outcomes of AS patients, including menses improvement, pregnancy outcome, endometrial thickness changes, and side effects in applying stem-cell based therapy, and every patient serves as their own control before stem cell therapy.

## 2. Materials and Methods

### 2.1. Search Strategy

This system review was performed according to the Preferred Reported Items for Systematic Reviews and Meta Analyses (PRISMA) guideline [15]. Two independent researchers (Y-M Zhao, Q-F Luo) systematically searched for the eligible studies on the database of PubMed, Embase, Web of Science, Cochrane, and Clinical Trials (published up to October 3, 2020). The search strategy was consisted of free word and Mesh terms: (a) “Stem Cell∗ [Mesh]” or “mesenchymal stromal cell∗” or “MSC∗” and (b) “Gynatresia [Mesh]” or “Asherman Syndrome” or “IUA” or “intrauterine adhesion”. In addition, other potential relevant studies were identified manually from references of eligible studies or reviews pertaining to this topic.

### 2.2. Selection Criteria

Studies were included based on the following criteria in accordance with PICOS. (a) Patients diagnosed with Asherman syndrome and have received conventional hysteroscopy adhesiolysis or hormone replacement treatment, but with no obvious alleviation. (b) Patients were administrated MSC-based stem cell therapy. (c) data containing base line after conventional treatment and outcomes concerning the MSC-based treatment. (d) Outcomes included menses improvement, endometrial thickness changes, side effect reports, or pregnancy outcome. (e) Internal control, prospective follow-up studies. (f) Written in English.

The exclusion criteria were as follows: (a) studies did not meet the inclusion criteria. (b) Written as an editorial, review, case report, clinical conference, and abstracts. (c) Involved nonhuman studies. (d) Repeated reports originating from the same database.

### 2.3. Data Extraction

Study selection and data extraction were carried out by two independent reviewers (Y-M Zhao, Q-F Luo). Any disagreement was discussed and submitted to the third reviewer (Y-F Qin) for confirmation. The extracted data from the eligible studies were as follows: author, year, country, patient number, age, etiology, symptoms, prior repair attempts, prior repair outcomes, IUA grade, MSC source, transplanted cell typed, cell number, transplanted section, hormonal therapy, and follow-up months. In addition, the outcome data were also extracted: menses improvement, endometrial thickness changes, side effects, and pregnancy outcome.

### 2.4. Outcome Measures

Menses improvement was defined as the changes of the volume or duration of the menses when compared with the baseline after the conventional therapy. Endometrial thickness was evaluated among 3-month post therapy, 6-month posttherapy, 9-month posttherapy, and baseline after the conventional therapy. Side effect reports were defined as the absolute number of the patients who complained uncomfortable after the treatments. Pregnancy outcome was decided by the number of patients who got pregnant after the MSC-based stem cell therapy or conventional therapy.

### 2.5. Quality Assessment

The risk of bias within the included studies was evaluated based on the criteria from “Assessing the Risk of Bias of Individual Studies in Systematic Reviews of Health Care Interventions” [16]. Detailed items in the checklist include selection bias, performance bias, attrition bias, detection, and reporting bias. Two reviewers (Q-F Luo, X Zhang), respectively, evaluated the eligible studies according to the constructed checklist. Any disagreement would be discussed and resolved by the third reviewer.

### 2.6. Statistical Analysis

This meta-analysis was conducted by the RevMan5.4 software (Cochrane Collaboration, London, United Kingdom). The indicators of menses improvement and pregnant outcome were displayed with the risk ratio (RR), with corresponding 95% confidential interval (CI). Briefly, the RR value was calculated as weighted averages by using a stratified analysis according to the Mantel-Haenszel Equations. As for the endometrial thickness, we used SMD and 95% CI to calculate the data. SMD was selected is because the enrolled studies applied for different measurement standard.

The chi-squared value and inconsistency index (*I*^2^) were used to assess the heterogeneity across each study. Specifically, the *Q* test obeys the chi-square (*χ*2) distribution with K-1 degrees of freedom. Therefore, after calculating the *Q* value, the probability could be obtained by chi-square analysis. In addition, the tau value is the estimated standard deviation of underlying effects across studies. RevMan presents an estimate of the between-study variance in a random-effects meta-analysis.

In the present study, a value of *P* < 0.1 or *I*^2^ > 50% was deemed with significant heterogeneity. Then, we adopt the random-effect model to analyze the data and performed subgroup analysis. Otherwise, we used the fixed-effect model. Moreover, subgroup analysis was conducted based on the follow-up period (half-month, 3-month, 6-month, and 9-month), with *P* < 0.05 indicating significant difference.

## 3. Results

### 3.1. Search Results

**Figure 1:**
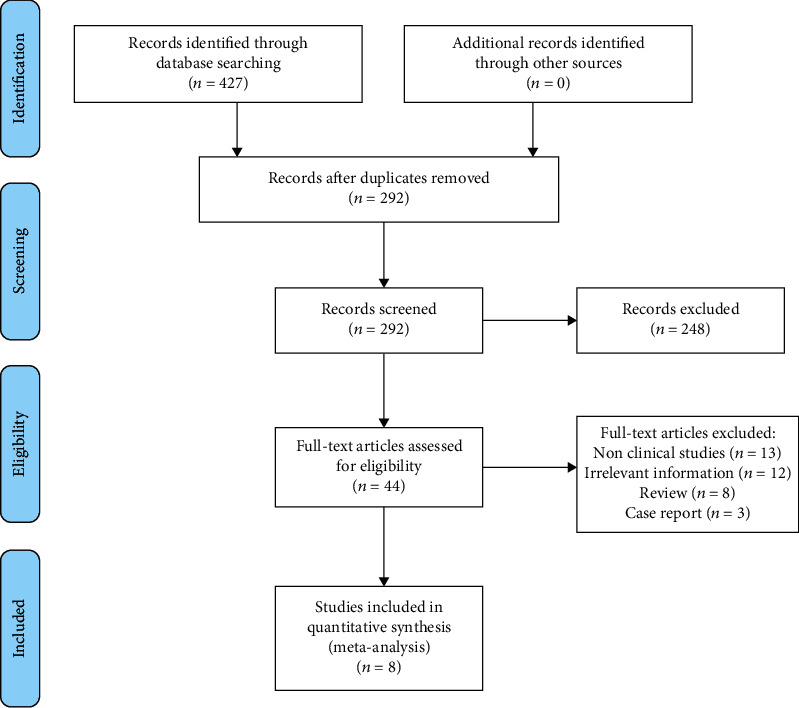
Flow diagram of the study selection process.

Our systematic search included 427 research articles according to the constructed searching strategy. The study selection process was shown in Figure 1. Briefly, a total of 135 duplicated articles were excluded in the process of importing the searching results to the Endnote software. Then, 248 studies were removed by reading the titles and abstracts, with the reason of nonrelevant to our study. Moreover, 44 articles were reread, and full text was screened according to the inclusion criteria, exclusion criteria, and data integrity. Lastly, we enrolled 8 clinical studies in our present systematic review [17–24].

### 3.2. Characteristics of Included Studies

We identified 8 studies (with data for 84 patients) in our analysis, and the basic characters of selected studies were summarized in Table 1. Specifically, the 8 trials were all published between 2014 and 2020. Patients' age ranges from 24 to 43. The main symptoms of patients are oligomenorrhea/amenorrhea and unsuccessful pregnancy. All patients failed to recover regularly normal menstruation and become pregnant after conventional treatment including hysteroscopic adhesiolysis, IUD, and hormone therapy.

Then, all these patients were recruited into the MSC-based treatment group, and the characters of MSC therapeutic strategy were shown in Table 2. Briefly speaking, three studies conducted marrow mononuclear stem cell-based therapy, while other stem cell sources are menstrual blood, umbilical cord, and adipose tissue. The appendix gives details about the specific stem cell number and transplantation section. After being treated by stem cells, all patients received hormone replacement therapy (HRT) to maintain the necessary level of estrogen. The outcome measures are menstruation improvement, endometrial thickness changes, and pregnancy outcome. Patients got pregnant naturally or humanly. As for the follow-up time, some studies did not report the specific data they acquired in six months or nine months, even though they follow up the patients. Thus, from our perspective, we encourage much more comprehensive data to be published, for establishing more solid evidence on stem cell-based therapy.

### 3.3. Risk of Bias

We next evaluated the potential risk bias by using the checklist as previously reported [16]. As shown in Table 3, nine questions, relating to the selection bias, performance bias, attrition bias, detection, and reporting bias, were analyzed in each enrolled study. In addition, we also assessed the likelihood of publication bias and heterogeneity for endometrial thickness changes by using the funnel plot and egger test, in which the selected results were less than 10. The funnel plot was roughly symmetric shape, suggesting less susceptibility to publication bias.

### 3.4. Outcome of Meta-Analysis

#### 3.4.1. Menstruation Improvement

**Figure 2:**
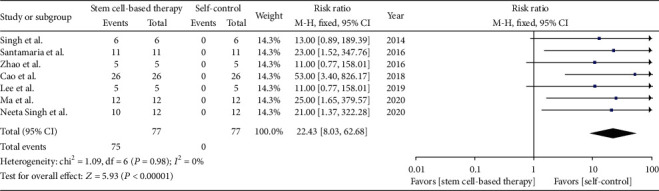
Menstruation improvement.

In our analysis of 7 trials (77 patients), almost all the patient treated by stem cell therapy reported improved menstruation in the first a few cycles, which was compared to the condition of menstruation before transplanting stem cells (Figure 2). We adopt count data in analyzing menstruation improvement, in which the improved prognosis were decided by the number of patients who reported with menstruation improvement; otherwise, it was recognized as zero. In addition, the figure also showed no statistically significant heterogeneity among included trials (*Q* test *P* > 0.1, *I*^2^ = 0%). However, one study reported some of the patients were with regression of menstruation in the third month compared to that of the first month after stem cell-based therapy [18].

#### 3.4.2. Pregnancy Outcome

**Figure 3:**
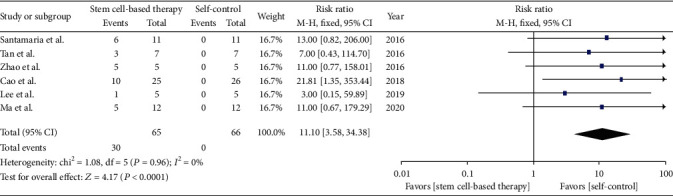
Pregnancy outcome.

To assess the pregnancy outcome, we conducted one analysis including 6 trials (66 patients). The analysis of pregnancy outcome showed that patients become pregnant naturally or humanly after they were treated by stem cells, while none of the patients had successful pregnancy with conventional treatment, including hysteroscopic adhesiolysis, IUD, and hormone therapy (Figure 3). The risk ratio is 11.1 with 95% CI [3.58, 34.38]. Meanwhile, there is no significantly statistical heterogeneity among included trials (*Q* test, *P* > 0.1, *I*^2^ = 0%).

#### 3.4.3. Endometrial Thickness Changes

**Figure 4:**
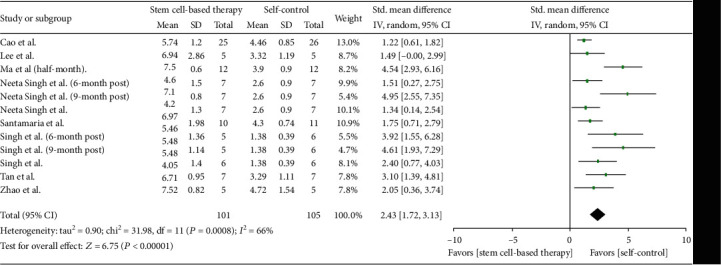
Endometrial thickness changes.

Eight studies (79 patients) recorded the endometrial thickness before and after patients received stem cell therapy (Figure 4). Before transplanting stem cells, the original endometrial thickness was served as self-control, for the reason that they had received conventional treatment, which include with hysteroscopic adhesiolysis, IUD, and hormone therapy. And after treatment with stem cell, the results showed that there was an increase of endometrial thickness in all eight studies, with significant between-study heterogeneity (*Q* test, *P* < 0.01, *I*^2^ = 66%).

**Figure 5:**
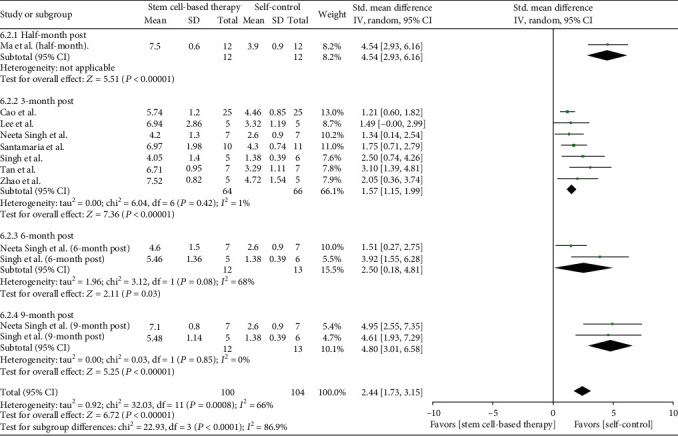
Subgroup analysis of endometrial thickness changes.

According to follow-up months, we conducted the subgroup analysis (Figure 5). Seven studies (66 patients) were included in the 3-month postanalysis, and the standardized mean difference is 1.57 (95% CI [1.15, 1.99]). We detected no statistical heterogeneity between studies (*Q* test, *P* > 0.1, *I*^2^ = 1%).

In the 6-month postanalysis, two studies (13 patients) were involved, and the standardized mean difference was 2.5 (95% CI [0.18, 4.81]). Although the heterogeneity between studies was statistically significant, both two trials showed a remarkable increase of endotmetrial thickness. The 9-month postanalysis including two studies (13 patients) showed that the standardized mean difference was 4.8 (95% CI [3.01, 6.58]), with no statistical heterogeneity (*Q* test, *P* > 0.1, *I*^2^ = 0%).

**Figure 6:**
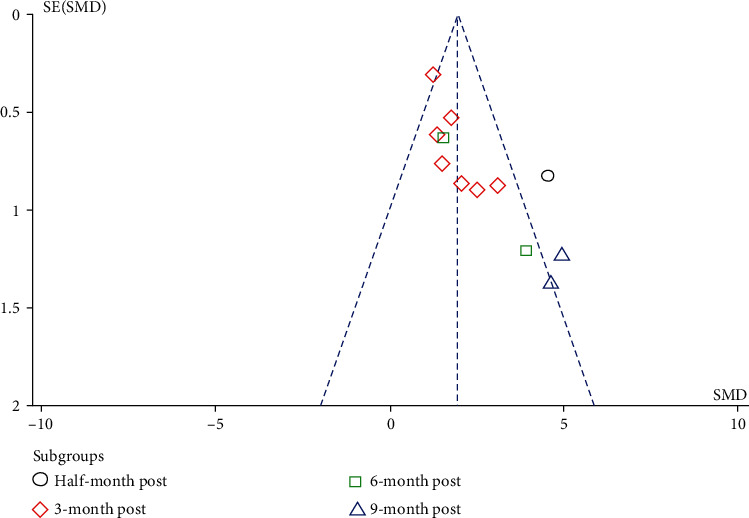
Funnel plot analysis.

In addition, we analyzed the publication bias and heterogeneity in endometrial thickness changes by using a funnel plot. As shown in Figure 6, the majority of 3-month poststudies lied inside the 95% CIs, with an even distribution around vertical, suggesting no obvious bias and heterogeneity. Moreover, 6-month studies were included into the 95% CIs. And both of the 9-month studies were beyond, which indicated that the follow-up time might be the source of heterogeneity, although there was a significant increase in the endotmetrial thickness 6 months or 9 months after the treatment (two studies). Much more long-term studies, such as at 6-month and 9-month time point, were warranted to evaluated the time effects in stem cell based treatment.

To further evaluate publication bias among the involved studies, we conducted the Egger test in the 3-month subgroup studies, in which there was shown with little heterogeneity. The Egger regression result suggests publication bias existing in the 3-month subgroup (bias = 2.262, *P* = 0.02). Then, we performed trim and fill analysis. After iterative calculation, it filled with four studies. However, the effect value did not change much (before trim: 1.765; after filling: 1.339), indicating that the previous result is rigid. However, we included 7 studies in the current analysis, which is not enough for 10 (Cochrane handbook). Therefore, we still look forward to more comprehensive clinical data to supplement and update the present meta-analysis.

### 3.5. Safety Evaluation

Of 8 studies (84 patients), 3 patients (3.57%) complained of experiencing adverse reactions in the process of the treatment, including loss of appetite, mild symptoms of gastritis, vomiting, and abdominal cramps. However, these symptoms disappeared subsequently. Of note, one of the clinical trials, conducted by Cao et al. [21], had assessed surgical complications, neutrophil percentage, c-reaction protein, and inflammation reaction in endometrial biopsies. They found no obvious adverse events occurring throughout the observation period in all 26 patients. Owning to no specific adverse reactions of the conventional therapeutic methods that were revealed in the included studies, we did not pool out the analysis here, but it is believed, from the present studies, that stem cell based therapy is safe and effective.

## 4. Discussion

This meta-analysis enrolled 8 clinical studies and included 84 patients, which is designed to compare the efficacy of stem cell-based therapy and traditional treatment. The results showed that stem-cell based therapy was safe and effective in improving Asherman syndrome patients' conditions. To be specific, those patients, benefited from stem cell based therapy, improved their menstrual volume, increased endometrial thickness, and restored regular menstrual cycles to some extent, which made it possible to provide a suitable environment for nurturing a baby [18–20]. Therefore, some of patients became pregnant naturally or humanly. Of note, in the combination of stem cell transplantation and hormone therapy, hormone was wildly recognized to play an adjuvant role in maintaining the necessary estrogen level, which is needed for restoring endometrium [25–27]. Thus, these results lend support to stem cell therapy as a therapeutic strategy that can enhance the prognosis of Asherman syndrome, especially for those failing conventional treatment.

When reviewing back the current clinical treatment strategies for the Asherman syndrome, it was greatly limited by the attainment of functional endometrium [8]. Previously, hysteroscopic adesiolysis was recognized as the preferred treatment method as it could help separating the fission of uterine, thus enlarging the uterine cavity for conceiving. However, there is a risk in inducing residual endometrium damage and accelerating scar formation in the surgical process [28, 29]. Additionally, hysteroscopic adhesiolysis was reported with the limit effect in treating unsuccessful reproduction [30]. It has been widely acknowledged that endometrial healing could be achieved in the presence of the high estrogen level [31]. The latest evidence has proven that hormone therapy could assist in restoring endometrium, but administrating hormone therapy alone has not been reported to improve the reproductive outcome in AS patients [32]. In this circumstance, stem cell therapy can be postulated as a novel strategy to recover the function of endometrium and enhance fertility, which brings hope for Asherman syndrome patients [27].

Findings from our meta-analysis reveal an overall beneficial effect of stem cell therapy for patients who have failed in conventional treatment, which include improved menstruation duration, enhanced pregnancy outcome, and increased endometrial thickness. These benefits are notable and inspiring. However, whether hormone treatment, following by the stem cell transplantation, played a major role in the combination therapy was proposed. Here, according to the selection criteria, all the patients enrolled in trails have received hysteroscopic adhesiolysis and hormone therapy previously, but has unsuccessful treatment outcome [21–23]. More importantly, every patient serves as their own control before and after transplanting stem cells. Thus, it is reasonable to explain the overall benefits that were majorly attributed to the stem cell therapy [33, 34]. These findings suggested that hormone therapy and stem cell transplantation would have a synergistic effect, rather than the antagonistic effect, in promoting alleviation of Asheman syndrome.

Our analysis has revealed robust and consistent findings supportive to the benefits achieved by administrating stem cell therapy, whereas there exists heterogeneity in the analysis of endometrial thickness. In order to solve this heterogeneity, we carried out subgroup analysis and found the follow-up months may be mainly responsible for the endometrial thickness changes. Moreover, we found there is no between-study heterogeneity in 3-month follow-up and 9-month follow-up groups, but the heterogeneity became most obvious in the 6-month follow-up group, suggesting that the time effect may be one of the reasons affecting endometrial recovery. However, in this condition, the increase of endometrial thickness is prominent when compared with the nonstem cell treatment period [17, 23]. Taken together, these studies support the generalisability of the observed beneficial effects of stem cell therapy in AS syndrome patients.

In terms of safety, no obvious and severe adverse reactions were observed during the process of stem cell therapy. There were 3 patients (3.57%) reported with lost appetite, mild gastritis, vomiting, or abdominal cramps, whereas these symptoms relieved subsequently. Moreover, none surgical complications, fever, and infection were broadcasted among the patients [21]. To ensure the invasiveness and safety, multiple methods have been reported to deliver stem cells to the endometrium, including attaching to the uterus wall, instilling into uterus fundus, subendometrial injection, and cervical instillation. These processes were believed atraumatic and causing smaller damage when compared to hysteroscopic adesiolysis [22]. In addition, none tumor genesis and tumor growth promotion cases were reported in applying stem cell therapy [35–37].

A limitation of this analysis is that the studies included are all self-control trials, which may be lack of blindness and randomization compared to random clinical trials. Secondly, although most of the studies selected in our analysis reported with encouraging results, there is still a potential risk of bias since some negative results of stem cell therapy in treating AS may not be published. Thirdly, there are differences in the source, number, and transmit sections of stem cells, in which lacking of normative standard. Thus, large scaling random clinical trials are highly needed to establish a unified rule of stem cell therapy. Finally, the ideal time of beginning this therapy remains unclear. Even though the enrolled trials generally concluded the results that patients, who have failed conventional treatment, could benefit from stem-cell based therapy, however, it raises a question that whether earlier implementation of stem cell would be much more beneficial.

## 5. Conclusion

This meta-analysis systematically reviewed and synthesized the outcomes of stem cell based therapy in treating Asherman syndrome, which suggest that stem cell and hormone combination therapy have superior therapeutic effects in improving menstruation duration, pregnancy outcome, and endometrial thickness. Moreover, this kind of therapy was also concluded with a favorable safety profile. Taken together, this meta-analysis provides an available evidence and wider perspective in the management of Asherman syndrome. However, further studies with large sample sizes are needed to establish more solid evidence for administrating this therapy in clinic.

## Figures and Tables

**Table 1:** Basic characters and primary treatment attempt for AS syndrome.

Authors	Country	Year	Number of patients	Age	Etiology	Symptoms	Prior repair attempts	Prior repair outcomes	IUA grade/score
Singh et al.	India	2014	6	29.8 ± 3.37	D&C, genital TB	Infertility, amenorrhea	Hysteroscopic adhesiolysis + HT (ALL)	Failed	IUA grade III-IV

Santamaria et al.	Spain	2016	11	38 ± 4.8	Hysteroscopic myomectomy, D&C, unexplained	Scant spotting, Amenorrhea	Reparative operative hysteroscopies + HT (8)	Failed	IUA grade I-III

Tan et al.	China	2016	7	33.7 ± 1.5	Curettages	Infertility	Adhesiolysis + IUD + HT (5)	Failed	IUA grade III-V

Zhao et al.	China	2016	5	33 ± 4.4	D&C	Infertility, Hypomenorrhea	Hysteroscopy adhesiolysis (all)	Failed	NA

Cao et al.	China	2018	26	35.1 ± 3.8	D&C	Infertility, Hypomenorrhea, amenorrhea	Hysteroscopy adhesiolysis (all)	Failed	IUA score 9.12 ± 1.51

Lee et al.	Korea	2019	5	39.2 ± 2.8	D&C, diagnostic laparoscopy, unexplained	Infertility	Hysteroscopy adhesiolysis + HT (all)	Failed	IUA grade III

Singh et al.	India	2020	12	27.86 ± 3.98	D&C, Cesarean section, genital TB, postpartum sepsis	Amenorrhea, Scanty spotting	Hysteroscopic adhesiolysis + HT (all)	Failed	IUA grade I-IV

Ma et al.	China	2020	12	36 ± 3.6	Curettage, infection, unexplained	Refractory IUA, infertility	Hysteroscopic adhesiolysis + HT (all)	Failed	IUA grade III-IV

AS: Asherman syndrome; D&C: dilation and curettage; TB: tuberculosis; HT: hormone therapy; IUD: intrauterine device; IUA: intrauterine adhesion; NA: not applicable.

**Table 2:** Characters of stem cell-based therapy in treating AS syndrome.

Authors/year	Cell source	Transplant cell type	Cell number (10^6^)	Transplanted section	HT	Follow up (months)
Singh et al. 2014	Bone marrow	Mononuclear stem cells	103.3 ± 20.45	Subendometrial transmyometrium injection (fundus, anterior, posterior)	Oral estradiol valerate, 6 mg/day (12 weeks). Medroxy progesterone (last 10 days). Patients (started menstruation): Shifted to cyclical oral estrogen valerate 2 mg (tid, day 1 to day 26), progesterone 10 mg daily (day 16 to day 25).	9

Santamaria et al. 2016	Per blood mobilization	CD133^+^ bone marrow Derived stem cells	123.6	Infusion into spiral arterioles	Hormonal replacement therapy (Progyluton™)	6

Tan et al. 2016	Menstrual blood	Menstrual blood Derived stromal cells	10^6^ (5 patients), 10^6^ × 2 (2 patients)	Instill into uterus fundus	MenSCs collection (day 5), oestradiol (4 mg, 14 day). MenSCs transplant, oestradiol (6 mg, 21 day). ET < 7 mm: progesterone injection 40 mg.	6

Zhao et al. 2016	Bone marrow	BM-mononuclear stem cells loaded in collagen scaffold	4 cm × 6 cm scaffold (5 × 10^6^ cm^2^)	Attach to the uterine wall	Progynova (6 mg, 10 days), operation, continuous Progynova (6 mg, 30 days), progesterone injection (60 mg, 30^th^ day)	3

Cao et al. 2018	Umbilical cord	Umbilical cord-derived Mesenchymal stromal cells	10	Attach to the uterine wall	Progynova (6 mg, 10 days), operation, continuous Progynova (6 mg, 30 days), progesterone injection (60 mg, 30^th^ day)	30

Lee et al. 2019	Adipose tissue	Adipose-derived MSCs loaded in stromal vascular fraction	4.6	Cervical instillation	Oral estradiol valerate (6 mg, day1 to day 25), Oral Medroxyprogesterone (10 mg, day 21 to day 25)	23

Singh et al. 2020	Bone marrow	BM-mononuclear stem cells	65.3 ± 37.2	Subendometrial transmyometrium injection (fundus, anterior, posterior)	Oral estradiol valerate (2 mg, Tid,12 weeks), ET > 6 mm: Medroxyprogesterone (10 mg, last 10 days in 12 week) ET < 6 mm: additional cycle.	60

Ma et al. 2020	Menstrual blood	Menstrual blood stem Cells (MenSCs)	10	Endometrium	Oral estradiol (2 mg, Tid, 14 days) + additional 3 days(ET < 7 mm)	NA

AS: Asherman syndrome; G-CSF: granulocyte colony-stimulating factor; BM: bone marrow; Tid: three times daily; ET: endometrium thickness.

**Table 3:** Checklist for quality assessment of the case series study.

Risk of bias	Criterion	Santamaria 2016	Singh 2014	Tan 2016	Cao. 2018	Singh 2020	Zhao 2016	Lee 2019	Ma 2020
Selection bias	Does the design or analysis control account for important confounding and modifying variables through matching, stratification, multivariable analysis, or other approaches?	Yes	Yes	Yes	Yes	Yes	Yes	Yes	Yes

Performance bias	Did researchers rule out any impact from a concurrent intervention or an unintended exposure that might bias results?	Yes	Yes	NA	NA	NA	Yes	NA	Yes
	Did the study maintain fidelity to the intervention protocol?	Yes	Yes	Yes	Yes	Yes	Yes	Yes	Yes

Attrition bias	If attrition (overall or differential nonresponse, dropout, loss to follow-up, or exclusion of participants) was a concern, were missing data handled appropriately (e.g., intention to treat analysis and imputation)?	Yes	Yes	Yes	Yes	NA	Yes	NA	Yes

Detection bias	Were the outcome assessors blinded to the intervention or exposure status of participants?	NA	NA	NA	NA	NA	NA	NO	NA
	Were interventions/exposures/assessed/defined using valid and reliable measures implemented consistently across all study participants?	Yes	Yes	Yes	Yes	Yes	Yes	Yes	Yes
	Were outcomes assessed/defined using valid and reliable measures implemented consistently across all study participants?	Yes	Yes	Yes	Yes	Yes	Yes	Yes	Yes
	Were confounding variable sassessed using valid and reliable measures implemented consistently across all study participants?	Yes	NA	NA	NA	NA	NA	NA	Yes

Reporting bias	Were the potential outcomes prespectified by the researchers? Are all prespecified outcomes reported?	Yes	Yes	Yes	Yes	Yes	Yes	Yes	Yes

## Data Availability

All data generated or analysed during this study are included in this published article.

## Supplementary Materials

Supplementary Materials Appendix S1: the PRISMA checklist of this meta-analysis.
